# Acupuncture Treatment for Hip Pain: A Systematic Review and Meta-Analysis

**DOI:** 10.3390/healthcare11111624

**Published:** 2023-06-01

**Authors:** Hyun Suk Park, Hye In Jeong, Soo-Hyun Sung, Kyeong Han Kim

**Affiliations:** 1 Department of Korean Medicine Rehabilitation, Bucheon Jaseng Hospital of Korean Medicine, Bucheon 14598, Republic of Korea; stevekingking@hanmail.net; 2 Department of Preventive Medicine, College of Korean Medicine, Kyung Hee University, Seoul 02447, Republic of Korea; frogcream@gmail.com; 3 Department of Policy Development, National Institute of Korean Medicine Development, Seoul 04554, Republic of Korea; 4 Department of Preventive Medicine, College of Korean Medicine, Woosuk University, Jeonju 54986, Republic of Korea

**Keywords:** acupuncture, hip pain, systemic review, meta-analysis, randomised controlled trials

## Abstract

Acupuncture treatment (AT) is an effective treatment for pain relief; however, there are few systematic reviews that have reported on the effectiveness of AT for hip pain. This systematic review aimed to evaluate the efficacy and safety of AT of hip pain. We searched eight databases for randomised controlled trials (RCTs) evaluating the effect of AT on hip pain until August 2022. Twelve RCTs (806 patients) were included: two reported a significant effect of AT compared with that of conventional medicine (CM) alone for hip pain; two reported significant effects of AT + CM compared with that of CM alone in terms of Visual Analogue Scale (VAS); two reported a significant effect of AT + CM compared with that of Sham AT + CM in terms of anaesthetic dosage; two reported a significant effect of AT + CM compared with that of Sham AT + CM in terms of the side effects associated with analgesic use; one study reported a significant effect of AT compared to that of no-treatment. No serious adverse events were reported. Our findings demonstrate the potential of AT in managing hip pain. Given the low quality and small sample sizes of the studies, the evidence supporting AT for hip pain management was weak. Further clinical trials and systematic reviews are required. The protocol of the current study was registered in the PROSPERO International prospective register of systematic reviews (CRD42017079586).

## 1. Introduction

The hip joint is a ball-and-socket joint composed of the femoral head and the acetabulum, making it one of the largest and most stable joints in the human body [1]. Despite its inherent stability, the hip joint provides the necessary high-degree mobility for performing daily activities such as standing and walking. The area surrounding the hip joint is supported by strong joint ligaments and a thick, strong muscle layer, which plays a vital role in stabilising the joint and providing mobility [2].

Hip pain refers to pain felt in or around the hip joint, which can result in functional deterioration, such as gait disturbance and limitations in performing activities of daily living. In addition, they can cause emotional pain. The causes of hip joint pain may include diseases of the hip joint, musculoskeletal system around the hip joint, soft tissue abnormalities, nerve entrapment, and damage [3].

Through a survey of 6500 adults aged 60 years or older in the United States, Christmas et al. revealed that 14.3% reported severe hip pain in the past six weeks. Treatment is necessary as it can reduce the ability to perform tasks and negatively impact the quality of life of patients and their families [4].

Conventional medical treatment for hip joint pain typically involves surgical or drug treatment, and sometimes hip replacement surgery to alleviate pain. However, Traditional Chinese Medicine (TCM) can be used as an alternative treatment method to avoid the side effects of conventional drugs and surgical treatments [5]. Kim et al. reported that TCM treatment methods for hip pain include acupuncture, pharmacopuncture, herbal medicine, cupping, moxibustion, and Chuna (Korean-style manual therapy), with the acupuncture points GB30, LR3, BL36, and ST31 [6].

Acupuncture is known to be effective for pain relief. The general mechanism of acupuncture treatment is to act on the central nervous system to stimulate the secretion of opioid peptides such as β-endorphin, enkephalin, and dynorphin, which produce analgesic effects [7,8]. In addition, there are various types of acupuncture, including auricular acupuncture, electroacupuncture, pharmacopuncture, and acutomy. Among them, auricular acupuncture is known to relieve pain by stimulating the nerves of the auricle and correcting imbalances in the body in both directions [9,10].

Studies on the effect of acupuncture on hip pain mainly consisted of case studies and randomised controlled trials (RCTs). While many TCM institutions have treated numerous patients with hip pain using acupuncture, relatively few studies have presented evidence of its effectiveness. Among the diseases that can cause hip pain, a systematic review of the effects of acupuncture has been conducted only in hip joint OA [5]. Therefore, this study conducted a systematic review of previous RCTs that used acupuncture in all diseases that cause hip pain including hip OA to explore and present evidence of its effects. 

## 2. Materials and Methods

The protocol of the current study was registered in the PROSPERO International prospective register of systematic reviews (CRD42017079586). This systematic review was conducted and is reported according to the Preferred Reporting Items for Systematic Reviews and Meta-Analyses (PRISMA) guidelines (2020).

### 2.1. Criteria for Inclusion and Exclusion of Studies

#### 2.1.1. Study Design

We included randomised controlled trials (RCTs) that assessed the efficacy of acupuncture in controlling hip pain. No language restrictions were imposed.

#### 2.1.2. Participants

Participants of any age or sex with hip pain were included. Patients with hip joint diseases (e.g., hip arthritis, hip osteoarthritis, and avascular necrosis of the femoral head) with hip pain were also considered for inclusion. Patients with hip problems but without pain were excluded.

#### 2.1.3. Interventions

We included studies using any type of acupuncture treatment (e.g., acupuncture, electroacupuncture, scalp acupuncture, or auricular acupuncture) as an intervention. We considered acupuncture only when needles were inserted into the skin. However, we excluded studies that used pharmacopuncture, transcutaneous electrical acupoint stimulation, or transcutaneous electrical nerve stimulator in the intervention group. 

#### 2.1.4. Comparisons

Comparisons of interventions, including placebo, sham acupuncture, no-treatment, and other active conventional treatments, were considered for inclusion. Conventional treatments were allowed if they were administered to both groups. However, when the control group underwent only a single treatment, we excluded the studies in which acupuncture was used, such as those that only compared different types of acupuncture.

#### 2.1.5. Outcomes

In this study, pain scores were considered as the primary outcome. Secondary outcomes included hip function and symptoms, quality of life, effectiveness rate for hip pain, requirement of Patient-Controlled Analgesia (PCA), and adverse events.

### 2.2. Search Strategy

We searched PubMed, Embase, the Cochrane Central Register of Controlled Trials (CENTRAL, The Cochrane Library), China National Knowledge Infrastructure (CNKI), Research Information Service System (RISS), Korean Studies Information Service System (KISS), Oriental Medicine Advanced Searching Integrated System (OASIS), and ScienceOn from database inception to August 2022. The full search terms and strategies are listed in Table S1.

### 2.3. Data Selection

From the studies obtained, two authors (HS and HI) individually chose studies to be included in the analysis after thoroughly reviewing the title, abstract, and main text. Once the authors independently ensured that the studies fulfilled the inclusion criteria, they cross-checked their findings. In case of any disagreements on the selected studies, a discussion was held with a third author (KH) to address the concerns and reach a consensus.

### 2.4. Data Extraction

From the studies that were finally selected, two researchers (HS and HI) independently investigated the authors, publication year, interventions, comparative interventions, sample size, outcomes, and results. Disagreements, if any, were resolved through discussion with a third researcher (KH).

### 2.5. Risk of Bias 

The chosen papers were assessed for Risk of Bias (ROB) by two researchers (HS and HI) using the evaluation criteria outlined in the Cochrane Handbook for Systematic Reviews of Interventions. The evaluation of ROB covered six domains for each paper, namely, sequence generation, allocation sequence concealment, blinding of participants and personnel, blinding of outcome assessment, incomplete outcome data, and selective outcome reporting. Both researchers (HS and HI) independently assigned a rating of low, uncertain, or high risk to each domain, and any discrepancies were resolved through discussions involving a third researcher (KH).

### 2.6. Meta-Analysis

The meta-analyses were conducted utilizing RevMan 5.4 (version 5.4 for Windows (Nordic Cochrane Centre, Copenhagen, Denmark). Continuous and dichotomous data were expressed as mean differences and odds ratios, respectively, with 95% confidence intervals.

## 3. Results

### 3.1. Study Selection and Description

**Figure 1:**
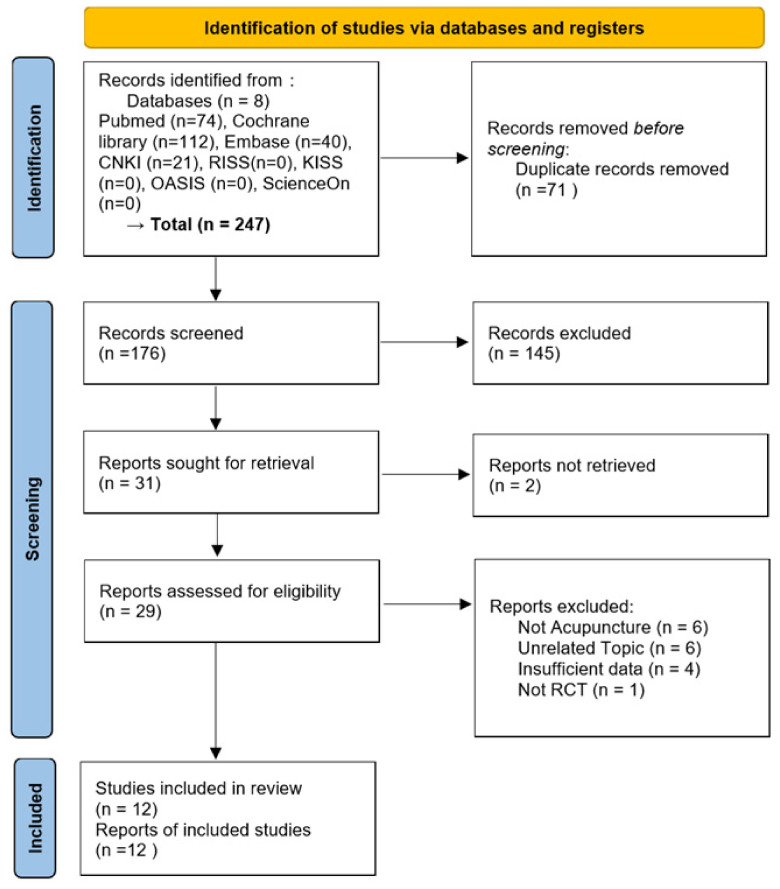
Flowchart of the RCT selection process.

After searching eight databases, 247 articles were retrieved: 74 from PubMed, 112 from the Cochrane Library, 40 from Embase, and 21 from CNKI. No articles were found in the RISS, KISS, OASIS, or ScienceOn databases. After excluding 71 overlapping studies, 176 papers were screened based on titles and abstracts, and 145 studies were excluded. Subsequently, the full texts of 31 papers that passed the initial screening process were reviewed, and six that did not use acupuncture as an intervention method, six that were irrelevant to the topic, four with insufficient data, and one that was not an RCT were excluded. Finally, 12 articles were selected, excluding two studies for which the full text could not be obtained. The study selection process is illustrated in Figure 1, following the Preferred Reporting Items for Systematic Reviews and Meta-Analyses guidelines. Table 1 summarises the details of the included studies.

### 3.2. Participants 

A total of 806 patients with hip pain were evaluated in 12 randomised controlled trials (RCTs), with 412 patients in the experimental group and 394 in the control group. Ten papers mentioned 598 people, of whom 262 were men and 336 were women.

### 3.3. Intervention

The intervention group was divided into two groups: Acupuncture Treatment (AT) [11,12,13,14,15,16] and AT + Conventional Medicine (CM) [17,18,19,20,21,22]. The characteristics of the intervention groups are summarised in Table 2.

#### 3.3.1. Acupuncture Points

ST31 [12,16], GB29 [11,12], GB30 [11,16], GB34 [11,16], and LI4 [11,14] were used in each of two different studies. Ashi points were also used in four studies [11,13,16,17]. MA-AH4 (hip joint) and MA-TF1 (shenmen) were used in all four studies using auricular acupuncture, [19,20,21,22] and MA-AH4 (hip joint) and MA-TF1 (shenmen) were used in three studies [19,20,22]. IC1 (lungs) and MA-AT1 (thalamus) were used in two studies [19,20]. GB31, GB43, ST44, ST36, ST40, LR10, LR11, EX28, LV3, BL37, BL54, SP10, MA-AT1 (subcortical), MA-SC (kidney), and 1 cun anterior to the mandibular angle (hip joint) were each used in one study.

#### 3.3.2. Needle Type (Diameter, Length)

The type of acupuncture used was specified in 11 studies. Needles of four diameters (0.18, 0.25, 0.30, and 0.40 mm) were used in six studies [11,12,14,16,17,18]. Needles with a diameter of 0.30 mm were used in three studies [14,16,17], and needles with diameter of 0.18 mm [18], 0.25 mm [11], and 0.40 mm [12] were used in each study. The needle length varied from 25 mm to 75 mm. All the studies that used auricular acupuncture used needles with a diameter of 0.22 mm. Three studies [19,20,22] used a needle with a length of 1.5 mm, and one study [21] used a needle with a length of 1.3 mm. One study [15] did not report the needle type, and another study [13] did not clearly indicate needle size.

#### 3.3.3. Depth of Insertion

Three studies [12,17,18] reported the needle depth. In two studies, acupuncture needles were inserted more than 30 mm at depths of 30–40 mm [12] and 30 and 50 mm, respectively, according to the acupoints. In another study [17], the insertion depth was relatively shallow at 5 to 10 mm.

#### 3.3.4. Angle of Insertion

Two studies [12,18] reported insertion angles of 90° (perpendicular to the skin).

#### 3.3.5. Needle Retention Time

The acupuncture retention time varied from 5 to 45 min [11,12,13,16]. Studies using auricular acupuncture have reported acupuncture retention times ranging from two days [22] to four days [19,21].

#### 3.3.6. Stimulation

Manual stimulation was applied along with acupuncture in three studies [11,13,15], and electrical stimulation was applied in two studies [12,14]. The electroacupuncture frequencies were 2/10 Hz [14] and 2/100 Hz [12].

### 3.4. Control Intervention

The control intervention was classified into four types: no-treatment [14,15], CM [11,12,13,17,18], Sham AT [16], and Sham AT + CM combined treatment [19,20,21,22]. In detail, AT was compared with no-treatment, and CM, Sham AT, and AT + CM combined treatment was compared with CM and Sham AT + CM combined treatment.

### 3.5. Intervention Effects

#### 3.5.1. Outcome Measures

The evaluation tools used in the included studies were diverse, with >20. Among them, the visual analogue scale (VAS) was used in seven studies [12,14,17,18,19,20,21], and the Harris score was used in three studies [12,17,21]. In addition, VAS Scores [11,15], piritramide requirement [19,22], time to first piritramide request [19,22], and fentanyl dose [20,22] were used as evaluation tools in two studies. All other evaluation tools were used in this study.

#### 3.5.2. Types of Disease 

In 12 RCTs, the diseases that caused hip joint pain were identified as Hip OA (Hip Osteoarthritis) in eight cases [11,12,14,15,16,19,20,22], hip fracture in two cases [17,18], avascular necrosis (AVN) of the hip in one case [21], and bursitis around the hip in one case [13]. In addition, six studies [11,18,19,20,21,22] performed total hip arthroplasty (THA), and THA was performed for hip OA in four [11,19,20,22] of the six studies.

#### 3.5.3. AT versus CM (Conventional Medicine)

Three studies [11,12,13] compared AT and CM treatments in patients who did not undergo THA, and two studies [11,13] showed that AT had a significant effect compared to CM treatment in patients with hip joint pain. In one study [11], AT showed a significant effect compared to CM treatment in relation to the WOMAC (Western Ontario and McMaster Universities Osteoarthritis Index) score (*p* < 0.05), and in another study [13], in relation to the VAS and Harris score (Pain, Function, ROM, Total), AT showed a significant effect on CM treatment and were considered significant (*p* < 0.05).

#### 3.5.4. AT + CM versus CM

**Figure 2:**
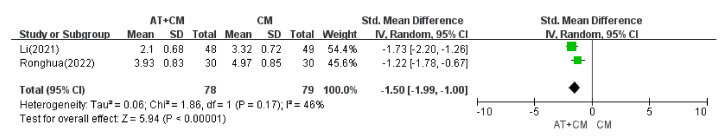
Meta-analysis (VAS). AT; Acupuncture Treatment CM; Conventional Medicine [17,18].

In two studies [17,18], the combination of AT and CM treatment was compared with CM treatment alone. One study [17] compared the combination of AT and CM treatment with CM treatment alone after PRFA surgery and one study [18] before THA. A meta-analysis was conducted on the VAS score. As a result of the meta-analysis, combined AT and CM treatment showed a statistically significant reduction in the VAS score compared to CM treatment (*p* < 0.05) (Figure 2). In addition, in one study [18], AT and CM combined treatment showed a significant effect compared to CM treatment in relation to Anaesthesia positioning time and Anaesthesia completion time (*p* < 0.05), and in another study [17], in terms of Harris score (Pain, Function) and Inflammatory factors (CRP, TNF-α), AT and CM combined treatment showed a significant effect compared to CM treatment (*p* < 0.05).

#### 3.5.5. AT + CM versus Sham AT + CM

**Figure 3:**
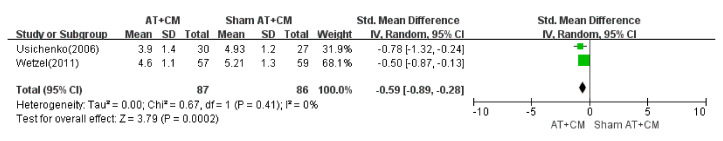
Meta-analysis (fentanyl dosage) [20,22].

**Figure 4:**
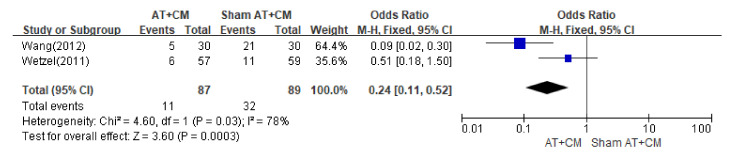
Meta-analysis (analgesic side effects). AT; Acupuncture Treatment CM; Conventional Medicine [21,22].

In four studies [19,20,21,22], the combined treatment of AT and CM was compared with the combined treatment of Sham AT and CM, and a meta-analysis was conducted on the anaesthetic dose and incidence of analgesic side effects (nausea and vomiting). As a result of the meta-analysis, in two studies [20,22], combined treatment of AT and CM showed statistically significant results in terms of fentanyl dosage compared with combined treatment of Sham AT and CM (*p* < 0.01) (Figure 3). In addition, two other studies [21,22] showed that AT and CM combined treatment had a statistically significant effect compared to Sham AT and CM combined treatment on the incidence of nausea and vomiting (OR 0.24; 95% CI 0.11 to 0.52; *p* = 0.0003) (Figure 4).

#### 3.5.6. AT versus No-treatment

In two studies [14,15], AT and no-treatment groups were compared and evaluated. In one study [10], statistically significant results were obtained for the VAS, T lymphocytes, and serological tests (*p* < 0.05). In the other study [15], WOMAC and SF-36 were used as evaluation indicators, but neither showed significant results.

#### 3.5.7. AT versus Sham AT

In one study [16], the AT and Sham AT groups were compared and evaluated, but no statistically significant effect was observed.

### 3.6. Adverse Events

AEs were reported in two study [15,19]. No AEs were reported in four studies [13,20,21,22], and AEs were not reported in seven studies [11,12,14,16,17,18]. In one study [15], minor local bleeding or hematoma, pain at the site of needle insertion, and vegetative symptoms were reported in the AT group and no AEs were reported in the no-treatment group. In another study [19], auricular haemorrhage, local pain, headache, and hip pain after needle placement were reported in the AT + CM group, and auricular haemorrhage and local pain were reported in the Sham AT + CM group.

### 3.7. Assessment for ROB

In nine of the 12 studies using random number tables, the item on the generation of random assignment order was evaluated as low risk. In three studies [15,17,21], there was no mention of the random assignment order method; therefore, they were evaluated as having an unclear risk.

In two studies [13,22], the group was assigned by an independent third party to conceal the assignment order; therefore, it was evaluated as low risk. In the remaining 11 studies, there was no mention of concealment of the assignment order; therefore, they were evaluated as having an unclear risk.

All 12 papers [11,12,13,14,15,16,17,18,19,20,21,22] were evaluated as high risk in terms of the blinding of subjects and researchers. Owing to the nature of RCTs on acupuncture, the risk of bias for subject and researcher blinding may be high.

In one study [16], the item on blinding of the outcome assessor was evaluated as high risk. Nine papers were evaluated as having unclear risk because they were not mentioned, and two papers [19,20] were evaluated as having low risk.

Two studies were missing values [16,20], and they were evaluated as high risk due to a lack of relevant comments. In one study [11], there were significant missing values in the patient group, which could have affected the results; therefore, this study was evaluated as high risk. Two studies [13,15] that did not address the exclusion/dropout outcomes were assessed as having unclear risks. The remaining seven papers were evaluated as low risk.

**Figure 5:**
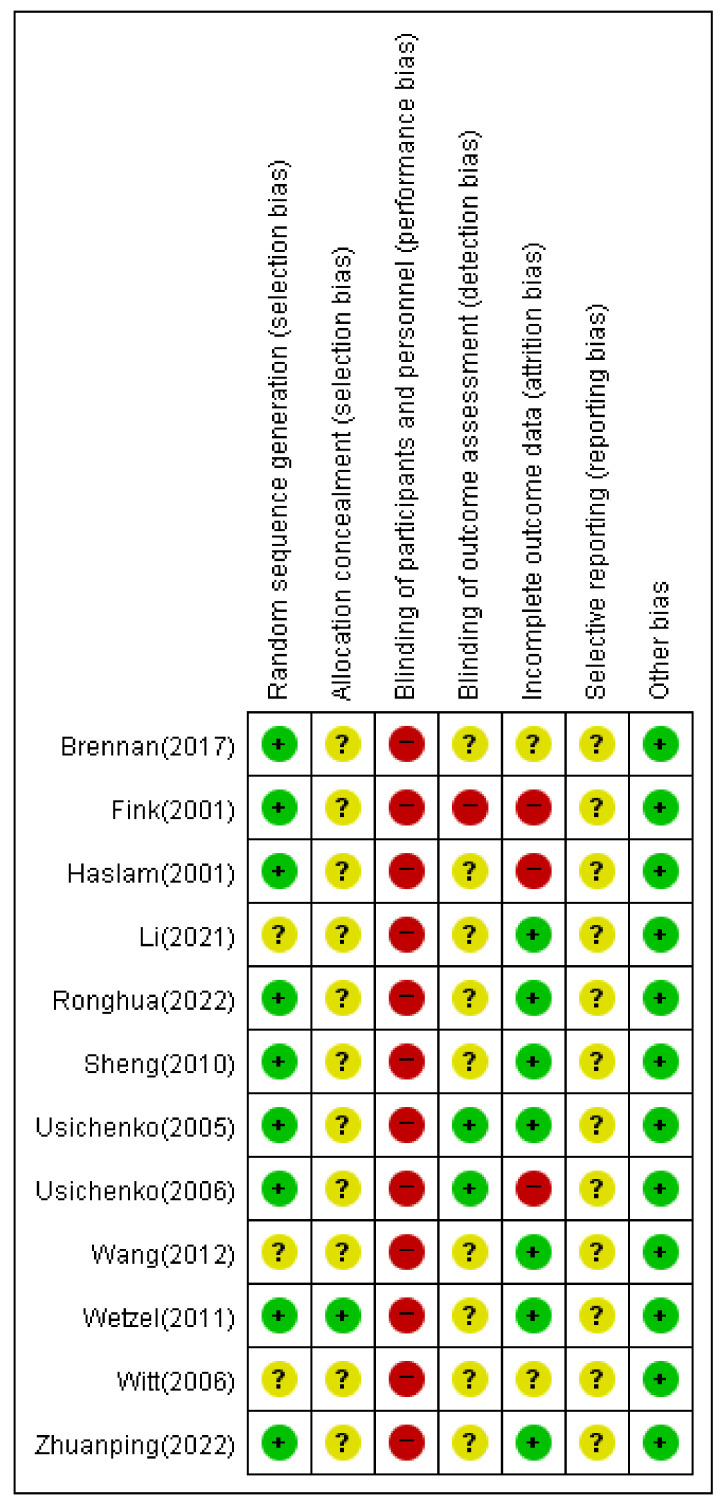
Risk of bias assessment [11,12,13,14,15,16,17,18,19,20,21,22].

**Figure 6:**
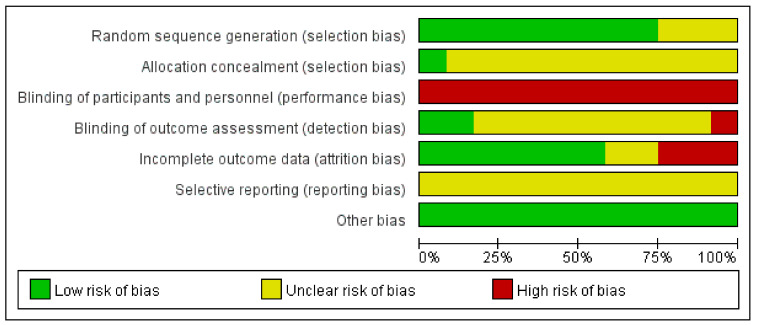
Risk of bias.

In all studies, protocols and preplanning were not mentioned and were assessed as having an uncertain risk of bias. All 12 studies were evaluated as having a low risk for other bias items. Figure 5 and Figure 6 summarize the details of the ROB for each RCT.

## 4. Discussion

Hip pain refers to pain inside or around the hip joint that can cause functional deterioration, such as gait disturbance and limitations in performing daily activities. Additionally, they can cause emotional distress. Hip pain can be caused by various factors, such as diseases of the hip joint, musculoskeletal system around the hip joint, soft tissue abnormalities, nerve entrapment, and damage [3].

In conventional medicine, surgical or drug treatment is typically used to treat hip pain, with total hip replacement surgery being a common option to alleviate the pain. However, in situations where the side effects of conventional medical drugs and surgical treatments are not small [23], acupuncture treatment among TCM treatments can be an alternative to conventional treatment, because it has few side effects and is effective in reducing pain [24,25].

Patients who do not experience symptom improvement with conventional medical treatments often seek treatment in TCM institutions. In TCM, hip pain is treated using acupuncture, electroacupuncture, herbal acupuncture, moxibustion, or Chuna therapy. Currently, many TCM institutions treat patients with hip pain using acupuncture, electroacupuncture, moxibustion, or Chuna [6].

Studies have shown that acupuncture has an analgesic effect by stimulating the secretion of opioid peptides such as β-endorphins, enkephalins, and dynorphins, which act in the central nervous system to produce analgesic effects [7,8]. However, no systematic literature review has examined the effectiveness of acupuncture in the treatment of hip pain. Therefore, in this study, a systematic literature review and meta-analysis of 12 RCT that studied the effect of acupuncture on hip pain were conducted to determine the effect of acupuncture on hip pain and to prepare the basis for prognostic judgment.

Eleven of the 12 studies reported the acupoints used, and the most used acupoints in studies using acupuncture were Ashi points, ST31, GB29, and GB30. It is believed that the Ashi points and ST31, GB29, and GB30 were commonly used because they are located around the hip joint. GB34 and LI4 were also used, and the reason for their use is considered to be because GB34 is a commonly used acupuncture point for musculoskeletal disorders throughout the body, and LI4 is effective for pain relief [26,27]. The most frequently used acupoints in studies using ear acupuncture are MA-AH4 and MA-TF1; it is thought that these two acupoints were also selected considering the effect of the acupuncture point and the location of pain.

Of the eight studies using standard acupuncture, four studies (50%) used needles longer than 50 mm, and two studies (66%) out of three studies reported a relatively deep insertion depth of 30 mm or more. This may be because the hip joint is anatomically composed of strong articular ligaments and a thick strong muscle layer around the joint [2]. Four of the studies used auricular acupuncture. The auricular acupuncture needle has a smaller diameter or length than general acupuncture needles but has a longer retention time. Auricular acupuncture is known to enhance the analgesic effect through continuous stimulation and is used for various types of pain treatment in clinical practice [9,10].

A previous systematic review (SR) included RCTs that used acupuncture to treat hip osteoarthritis (OA), but it did not arrive at favourable conclusions regarding acupuncture’s efficacy in treating hip pain caused by osteoarthritis [5]. This study aimed to investigate the effectiveness of acupuncture in all diseases that cause hip pain, including hip OA. Additionally, studies on pain occurring before and after total hip arthroplasty (THA) were also included, similar to previous studies conducted by Li [28] and Shin [29]. Nevertheless, this study is significant because only two studies [19,22] overlapped with Li’s study, and Shin’s study has not yet been conducted as a protocol.

We reviewed 12 RCTs to evaluate the effectiveness of acupuncture in the treatment of hip pain. While some studies compared acupuncture alone to combined treatment with acupuncture and CM, most of the RCTs we analysed involved combined treatment, mainly because of specific circumstances, such as before and after surgery. Our analysis of the outcome measures revealed that acupuncture alone had a positive impact, whereas combined treatment demonstrated a greater effect. Notably, two RCTs, Zhuanping et al. and Witt et al., which had no-treatment applied to the control group, focused on simple hip osteoarthritis or pre-total hip arthroplasty.

Although immediate pain relief is crucial, it is equally important to consider pain duration. Therefore, most studies included in our analysis conducted follow-up assessments that varied from as short as 6 h to as long as 6 months. In some cases, where no effect was observed initially, such as in the study by Fink et al., no effect was observed in follow-up assessments. However, in studies by Li et al., Wang et al., and Zhuanping et al., in which no effect was observed initially, a positive effect was observed after a certain period. In contrast, Haslam et al. and Sheng et al. reported an immediate effect that lasted for some time. Acupuncture has been used to treat various pain conditions, including acute and chronic lower back pain, osteoarthritis, headache, and neck pain.

A meta-analysis was difficult to conduct because most treatments were performed under special conditions, and the diseases and types of treatments were diverse. Therefore, we conducted a meta-analysis of only a limited number of RCTs [17,18,20,22]. The meta-analysis compared the CM and sham acupuncture plus CM groups to the acupuncture plus CM group and showed statistically significant results in VAS reduction in the former and fentanyl dose in the latter. However, these results should be interpreted with caution.

Several adverse events were reported in two studies [15,19]. In one study of acupuncture [15], side effects such as minor local bleeding or hematoma, pain at the site of needle insertion, and vegetative symptoms were reported in the experimental acupuncture group. In one study of auricular acupuncture [19], side effects such as auricular haemorrhage, local pain, headache, and hip pain after needle placement were reported in both the experimental acupuncture group and the control sham acupuncture group. These side effects were treated appropriately without further complications. Similar side effects have been reported in other studies related to acupuncture [30,31] and auricular acupuncture [32]; therefore, they can be considered common side effects of auricular acupuncture. One of the greatest advantages of acupuncture is its low incidence of side effects [33]; no fatal side effects were reported in the 12 RCTs included in this study.

Our study had several limitations. Most of the included studies had low methodological quality in the Cochrane ROB assessment. Among the selection biases, random sequence generation showed a low risk in approximately three-quarters of studies, due to the nature of acupuncture, blinding of participants, practitioners, and outcome assessors was not performed in all RCTs.

In the four RCTs that compared acupuncture with sham acupuncture, the sham acupuncture control interventions were judged credible; however, each sham acupuncture intervention was also judged to have a risk of weak acupuncture-specific effects due to the placement of nonpenetrating needles at the correct acupuncture points in one RCT and the use of penetrating needles not inserted at the correct points in the other RCT. One RCT did not report the accurate position.

In the meta-analysis of the study presented to us for assessment, it shows that there are several heterogeneous due to different AT points, AT sessions, AT needles (e.g., size, thickness, depth, and application site), assessment tools (e.g., VAS, WOMAC and SF-36), conventional medical treatment (e.g., used drugs, dosages and administration mode), and randomisation methods. Thus, the results of meta-analysis are insufficient information for a recommendation of treatment with acupuncture in patients with hip pain in its different causes. RCTs in specific pathologies with homogeneous protocols are needed to assess whether acupuncture is effective for the control or improvement of pain and other parameters in patients with hip pain.

## 5. Conclusions

This study analysed the effectiveness of acupuncture in treating various conditions that can cause hip pain. The research showed that combined treatment of AT and CM had a significant effect compared to CM alone or combined treatment of sham AT and CM. However, it is important to interpret the results carefully, because the study involved cases of combination of AT and CM, not AT alone.

We hope that our data can be used for further research and clinical practice in acupuncture treatment for hip pain, and we believe that high-quality studies are needed in this field.

## Figures and Tables

**Table 1:** Characteristics of the included studies.

Author (1st)	Year	Study Design	Types of Disease (Condition)	Sample Size, Gender (M/F)	Interventional Group (No. of Participants Analysed/Randomised)	Control Group (No. of Participants Analysed/Randomised)	Outcome Measures	Main Results	AE
Haslam [11]	2001	RCT	Hip OA; Awaiting total hip arthroplasty	32 (16/16)	(A) AT (16/16)	(B) CM (12/16)	WOMAC Scores (Post, 8 wks F/U)	Postitive ^a^ (Post, 8 wks F/U)	NR
Sheng [12]	2010	RCT	Hip OA	60 (23/37)	(A) AT (30/30)	(B) CM (30/30)	1. VAS 2. Harris score 2-1. Pain 2-2. Function 2-3. ROM 2-4. Total	1. Positive ^a^ 2-1. Positive ^a^ 2-2. Positive ^a^ 2-3. NS 2-4. Positive ^a^	NR
Brennan [13]	2017	Non-inferiority RCT	Greater Trochanteric Pain Syndrome (greater trochanteric or subgluteal bursitis)	50 (21/22) *	(A) AT (21/25)	(B) CM (22/25)	1. NRS (base, >1 wk, >3 wks, >6 wks) 2. PSFS (Weighted average)(base, >1 wk, >3 wks, >6 wks)	1. NS (base, >1 wk, >3 wks, >6 wks) 2. NS (base, >1 wk, >3 wks, >6 wks)	None
Zhuanping [14]	2022	RCT	Before THA	74 (41/33)	(A) AT (37/37)	(B) No-treatment (37/37)	1. VAS (>6 h, >12 h, >24 h) 2. T lymphocytes 2-1. CD3+ (>24 h) 2-2. CD4+ (>24 h) 2-3. CD8+ (>24 h) 2-4. CD4+/CD8+ (>24 h) 3. Serological test 3-1. IL-1β (>24 h) 3-2. TNF-α (>24 h) 3-3. ACTH (>24 h)	1. Positive ^a^ (>6 h, >12 h, >24 h) 2-1. Positive ^a^ (>24 h) 2-2. Positive ^a^ (>24 h) 2-3. Positive ^a^ (>24 h) 2-4. Positive ^a^ (>24 h) 3-1. Positive ^a^ (>24 h) 3-2. Positive ^a^ (>24 h) 3-3. Positive ^a^ (>24 h)	NR
Witt [15]	2006	RCT	Hip OA	92 (51/41)	(A) AT (51/51)	(B) No-treatment (41/41)	1. WOMAC 2. SF-36	1. NS 2. NS	(A) Minor local bleeding or hematoma, Pain at the site of needle insertion, Vegetative symptoms, Other (B) None
Fink [16]	2001	RCT	Coxarthrosis (Hip OA)	65 (43/22)	(A) AT (33/33)	(B) Sham AT (32/32)	1. Hip function (Lequesne) 2. Pain (VAS) 3. Overall assessment (Clarsson)	1. NS (>2 wks, >6 wks, >6 mths) 2. NS (>2 wks, >6 wks, >6 mths) 3. NS (>6 wks, >6 mths)	NR
Li [17]	2021	RCT	After PFNA due to intertrochanteric fracture	97 (48/49)	(A) AT + CM (48/48)	(B) CM (49/49)	1. VAS (>1 d, >3 d, >5 d, >7 d) 2. Harris score 2-1. Pain (>2 mth) 2-2. Function (>2 mth) 2-3. Deformity (>2 mth) 2-4. ROM (>2 mth) 3. MBI score (>2 mth) 4. Bone metabolism indexes 4-1. β-CTx (>2 mth) 4-2. PINP (>2 mth) 5. Inflammatory factors (>2 mth) 5-1. CRP 5-2. TNF-α	1. NS (>1 d, >3 d), Positive ^a (^>5 d, >7 d) 2-1. Positive ^a (^>2 mth) 2-2. Positive ^a (^>2 mth) 2-3. NS (>2 mth) 2-4. Positive (>2 mth) 3. Positive ^a (^>2 mth) 4-1. NS (>2 mth) 4-2. NS (>2 mth) 5-1. Positive ^a (^>2 mth) 5-2. Positive ^a (^>2 mth)	NR
Ronghua [18]	2022	RCT	Before Surgery Due to hip fracture	60 (17/43)	(A) AT + CM (30/30)	(B) CM (30/30)	1. VAS 1-1. Entering 1-2. Before positioning 1-3. After positioning 2. Anaesthesia-related time indicators 2-1. Anaesthesia positioning time 2-2. Anaesthesia completion time 2-3. Temperature sense disappearance time	1-1. NS 1-2. Positive ^a^ 1-3. Positive ^a^ 2-1. Positive ^a^ 2-2. Positive ^a^ 2-3. NS	NR
Usichenko [19]	2005	RCT	After THA due to hip OA	61 (26/35)	(A) AT + CM (29/31)	(B) Sham AT + CM (25/30)	1. Intraoperative fentanyl requirement 2. Time to first piritramide request 3. Piritramide requirement (>36 h, 36 h/kg, Total, Total/kg) 4. VAS 5. Total ibuprofen	1. NS 2. Positive ^a^ 3. Positive ^b (^>36 h, >36 h/kg, Total, Total/kg) 4. NS 5. NS	(A) Auricular hemorrhage, Local pain, Headache, Hip pain after needles (B) Auricular hemorrhage, Local pain
Usichenko [20]	2006	RCT	During THA due to hip OA	64 (28/29) *	(A) AT + CM (30/33)	(B) Sham AT + CM (27/31)	Fentanyl requirement adjusted to weight	Positive ^b^	None
Wang [21]	2012	RCT	After THA due to AVN	60 (23/37)	(A) AT + CM (30/30)	(B) Sham AT + CM (30/30)	1. VAS (>12 h, >24 h, >36 h, >48 h, >3 d, >4 d, >5 d, >7 d) 2. Dosage of analgesia (<12 h, 12–24 h, 24–36 h, 36–48 h) 3. Harris Score (>2 wks, >3 mths)	1. NS (>12 h, >36 h, >24 h, >48 h), Positive ^a (^>3 d, >4 d, >5 d, >7 d) 2. NS 3. NS (>3 mths), Positive ^a (^>2 wks)	None
Wetzel [22]	2011	RCT	During THA due to hip OA	120 (50/70)	(A) AT + CM (57/60)	(B) Sham AT + CM (59/60)	1. Fentanyl amount adjusted to body mass (ug/kg) 2. Time to first piritramide request 3. Piritramide requirement	1. Positive ^b^ 2. NS 3. NS	None

AE: adverse event; NR: not recorded; NS: no significant difference; ^a^: *p* < 0.05; ^b^: *p* < 0.01; Hip OA; Hip Osteoarthritis, THA: Total Hip Arthroplasty; WOMAC: Western Ontario and McMaster Universities Osteoarthritis Index; VAS: Visual analogue scale; ROM: Range of Motion; NRS: Numeral Rating Scale; PSFS: Patient-Specific Functional Scale; RCT: Randomised Controlled Trials; PINP: procollagen type I N-propeptide; CRP: C-Reactive Protein; TNF: tumour necrosis factor; AVN; avascular necrosis; AT: Acupuncture Treatment; CM: Conventional Medicine; * The gender of withdrawn people is not clear.

**Table 2:** Characteristics of acupuncture interventions in the included studies.

Author (1st)	Year	Regimen	Acupuncture Points	Type of Needle (Diameter, Length)	Depth of Insertion	Angle of Insertion	Needle Retention Time	Stimulation
Haslam [11]	2001	6 sessions (once a wk for 6 wks)	GB29, GB30, GB34, GB43, ST44, LI4, Ashi points *	GB30: 0.25 mm, 40 mm Etc: 0.25 mm, 25 mm	NR	NR	25 min	Manual stimulation of acupuncture
Sheng [12]	2010	8 sessions (2 times a wk for 4 wks)	ST31, GB29, LR10, LR11	0.40 mm, 75 mm	30~40 mm	90°	45 min	EA (2 Hz/100 Hz)
Brennan [13]	2017	3~7 sessions (for 6 wks)	Ashi points *	0.30~0.50 mm, 50~100 mm	NR	NR	5~7 min	Manual stimulation of dry needling
Zhuanping [14]	2022	Once	LI4, LV3, ST36, EX28	0.30 mm, 40 mm	NR	NR	NR	EA (2 Hz/10 Hz)
Witt [15]	2006	15 sessions (for 3 mth)	NR	NR	NR	NR	NR	Manual stimulation of acupuncture
Fink [16]	2001	9 sessions (for 3 wks)	BL37, BL54, ST31, ST40, GB30, GB31, GB34, Ashi points *	0.30 mm, 60 mm	NR	NR	30 min	NR
Li [17]	2021	7 sessions (daily for 7 days)	SP10, Ashi points *	SP10: 0.30 mm, 50 mm Ashi points: 0.30 mm, 75 mm	SP10: 30 mm Ashi point: 50 mm	NR	NR	EA
Ronghua [18]	2022	Once	1 cun anterior to mandibular angle (hip joint)	0.18 mm, 30 mm	5~10 mm	90°	NR	NR
Usichenko [19]	2005	Once	MA-AH4 (hip joint), MA-TF1 (shenmen), MA-IC1 (lung), MA-AT1 (thalamus)	0.22 mm, 1.5 mm (AA)	NR	NR	4 days	NR
Usichenko [20]	2006	Once	MA-AH4 (hip joint), MA-TF1 (shenmen), MA-IC1 (lung), MA-AT1 (thalamus)	0.22 mm, 1.5 mm (AA)	NR	NR	NR	NR
Wang [21]	2012	4 sessions (once for 4 days)	MA-AH4 (hip joint), MA-TF1 (shenmen), MA-AT1 (subcortex), MA-SC (kidney)	0.22 mm, 1.3 mm (AA)	NR	NR	4 days	NR
Wetzel [22]	2011	Once	MA-AH4 (hip joint), MA-TF1 (shenmen), MA-IC1 (lung)	0.22 mm, 1.5 mm (AA)	NR	NR	2 days	NR

* Ashi point: acupuncture point without a specific name or definite location, the site of which is determined by tenderness or other pathological responses; AA: Auricular Acupuncture; NR: not reported; wks: weeks; mth; months.

## Data Availability

The data presented in this study are available on request from the corresponding author (K.H.K).

## Supplementary Materials

The following supporting information can be downloaded at: https://www.mdpi.com/article/10.3390/healthcare11111624/s1, Table S1: Search strategy.

## Abbreviations

TCM: Traditional Chinese Medicine, RCTs: randomised controlled trials, TEAS: transcutaneous electrical acupoint stimulation, TENS: transcutaneous electrical nerve stimulator, CNKI: China National Knowledge Infrastructure, RISS: Research Information Service System, KISS: Korean Studies Information Service System, OASIS: Oriental Medicine Advanced Searching Integrated System, ROB: Risk of Bias, AT: Acupuncture Treatment, CM: Conventional Medicine, VAS: Visual analogue scale, hip OA: Hip Osteoarthritis, AVN: avascular necrosis, THA: total hip arthroplasty, WOMAC: Western Ontario and McMaster Universities Osteoarthritis Index, SR: systematic review.
