# Supplementary material for: Acupuncture Treatment for Hip Pain: A Systematic Review and Meta-Analysis
Source: Healthcare (Basel). 2023 Jun 1;11(11):1624. doi: 10.3390/healthcare11111624 (PMC10252336; doi:10.3390/healthcare11111624)
Supplement: Supplementary file 1 [file healthcare-11-01624-s001.zip › healthcare-2358819-supplementary.pdf]

Hip pain SR

Table S1. Search strategy in PubMed.

NO.	Search strategy	Item
#1.	Search Acupuncture[Mesh]	28599
#2	Search (acupuncture[TIAB]) OR (electroacupuncture[TIAB]) OR (acupoint[TIAB]) OR (auricular acupuncture[TIAB]) OR (ear acupuncture[TIAB]) OR (scalp acupuncture[TIAB]) OR (hand acupuncture[TIAB]) OR (pharmacoacupuncture[TIAB])	30152
#3	#1 OR #2	35938
#4	Search hip[Mesh] OR hip joint[Mesh] OR hip[TIAB] OR hip joint[TIAB]	169049
#5	Search pain[TIAB] OR ache[TIAB] OR disorder[TIAB] OR arthritis[TIAB] OR arthralgia[TIAB] OR arthropathy[TIAB] OR arthrophy[TIAB] OR osteoarthritis[TIAB] OR fracture[TIAB] OR dislocation [TIAB] OR tendinitis[TIAB] OR tendinopathy[TIAB] OR avascular necrosis of femoral head[TIAB] OR strain[TIAB] OR bursitis[TIAB] OR synovitis[TIAB] OR labrum tear[TIAB] OR impingement syndrome[TIAB] OR iliotibial band syndrome[TIAB] OR piriformis syndrome[TIAB] OR snapping hip syndrome[TIAB]	2253413
#6	#4 AND #5	74060
#7	Search “Randomized Controlled Trial”[PT] OR “Controlled Clinical Trial”[PT] OR randomized[TIAB] OR placebo[TIAB] OR “Clinical Trials as Topic”[Mesh: noexp] OR randomly[TIAB] OR trial[TI]	1556184
#8	#3 AND #6 AND #7	74

Table S2. Search strategy in cochrane library

NO.	Search strategy	Item
#1	MeSH descriptor: [Acupuncture] explode all trees	164
#2	(acupuncture):ti,ab,kw OR (electroacupuncture):ti,ab,kw OR ("acupoint"):ti,ab,kw OR ("auricular acupuncture"):ti,ab,kw OR ("ear acupuncture"):ti,ab,kw OR ("scalp acupuncture"):ti,ab,kw OR ("hand acupuncture"):ti,ab,kw OR ("pharmacoacupuncture"):ti,ab,kw	18758
#3	#1 OR #2	18758
#4	MeSH descriptor: [Hip]	446
#5	(hip):ti,ab,kw OR (hip joint):ti,ab,kw	26012
#6	(pain):ti,ab,kw OR (ache):ti,ab,kw OR (disorder):ti,ab,kw OR (arthritis):ti,ab,kw OR (arthralgia):ti,ab,kw OR (arthropathy):ti,ab,kw OR (arthrophy):ti,ab,kw OR (osteoarthritis):ti,ab,kw OR (fracture):ti,ab,kw OR (dislocation):ti,ab,kw OR (tendinitis):ti,ab,kw OR (tendinopathy):ti,ab,kw OR (avascular necrosis of femoral head):ti,ab,kw OR (strain):ti,ab,kw OR (bursitis):ti,ab,kw OR (synovitis):ti,ab,kw OR (labrum tear):ti,ab,kw OR (impingement syndrome):ti,ab,kw OR (iliotibial band syndrome):ti,ab,kw OR (piriformis syndrome):ti,ab,kw OR (snapping hip syndrome):ti,ab,kw	340918
#7	#4 OR #5	26012
#8	#6 AND #7	13561
#9	("Randomized Controlled Trial"):pt OR (“Controlled Clinical Trial”):pt OR (randomized):ti,ab,kw OR (placebo):ti,ab,kw OR (randomly):ti,ab,kw OR (trial):ti,ab,kw	1466249
#10	#3 AND #8 AND #9	112

supplementary table S3. search strategy in embase

NO.	Search strategy	Item
#1	'acupuncture'/exp	63466
#2	Search acupuncture:ti,ab,kw OR electroacupuncture:ti,ab,kw OR acupoint:ti,ab,kw OR 'auricular acupuncture':ti,ab,kw OR 'ear acupuncture':ti,ab,kw OR 'scalp acupuncture':ti,ab,kw OR 'hand acupuncture':ti,ab,kw OR 'pharmacoacupuncture':ti,ab,kw	42484
#3	#1 OR #2	63765
#4	'hip'/exp OR 'hip joint'/exp	125742
#5	pain:ti,ab,kw OR ache:ti,ab,kw OR disorder:ti,ab,kw OR arthritis:ti,ab,kw OR arthralgia:ti,ab,kw OR arthropathy:ti,ab,kw OR osteoarthritis:ti,ab,kw OR fracture:ti,ab,kw OR dislocation:ti,ab,kw OR tendinitis:ti,ab,kw OR tendinopathy:ti,ab,kw OR 'avascular necrosis of femoral head':ti,ab,kw OR strain:ti,ab,kw OR bursitis:ti,ab,kw OR synovitis:ti,ab,kw OR 'labrum tear':ti,ab,kw OR 'impingement syndrome':ti,ab,kw OR 'iliotibial band syndrome':ti,ab,kw OR 'piriformis syndrome':ti,ab,kw OR 'snapping hip syndrome':ti,ab,kw OR arthrophy:ti,ab,kw	3056280
#6	#4 AND #5	43476
#7	'randomized controlled trial'/exp	724261
#8	'randomized controlled trial':it OR 'controlled clinical trial':it OR randomized:ti,ab,kw OR placebo:ti,ab,kw OR randomly:ti,ab,kw OR trial:ab,ti OR 'clinical trials':ti,ab,kw	2268545
#9	#7 OR #8	2377689
#10	#3 AND #6 AND #9	40

Supplementary Table S4. Search strategy in CNKI

NO .	Search strategy	Item
#1	针 OR 针刺	118588
#2	电针 OR 耳针 OR 头皮针 OR 手针 OR 药针	38912
#3	#1 OR #2	299482
#4	SU=(针) OR SU=(针刺) OR SU=(电针) OR SU=(头皮针) OR SU=(手针) OR SU=(药针) SU=(acupuncture) OR SU=(electroacupuncture) OR SU=(auricular acupuncture) OR SU=(ear acupuncture) OR SU=(scalp acupuncture) OR SU=(hand acupuncture) OR SU=(pharmacoacupuncture)	226977
#5	#3 OR #4 SU=(针) OR SU=(针刺) OR SU=(电针) OR SU=(头皮针) OR SU=(手针) OR SU=(药针) OR SU=(acupuncture) OR SU=(electroacupuncture) OR SU=(auricular acupuncture) OR SU=(ear acupuncture) OR SU=(scalp acupuncture) OR SU=(hand acupuncture) OR SU=(pharmacoacupuncture)	368953
#6	髋关节疼痛 OR 髋关节紊乱 OR 髋关节脱位 OR 髋关节病 OR 髋关节骨性关节炎 OR 髋关节滑膜炎 OR 股骨头缺血性坏死 OR 髋部骨折 OR 髋关节撞击综合征 OR 髂胫束综合征 OR 梨状肌综合征 OR 弹响髋综合征 OR 髋关节盂唇撕裂 OR 髋关节置换术	33682
#7	hip pain OR hip disorder OR hip dislocation OR hip arthropathy OR hip osteoarthritis OR hip synovitis OR avascular necrosis of femoral head OR hip fracture OR hip impingement syndrome OR iliotibial band syndrome OR piriformis syndrome OR snapping hip syndrome OR hip labrum tear OR hip arthrophy	21332
#8	#6 OR #7 SU=(髋关节疼痛) OR SU=(髋关节紊乱) OR SU=(髋关节脱位) OR SU=(髋关节病) OR SU=(髋关节骨性关节炎) OR SU=(髋关节滑膜炎) OR SU=(股骨头缺血性坏死) OR SU=(髋部骨折) OR SU=(髋关节撞击综合征) OR SU=(髂胫束综合征) OR SU=(梨状肌综合征) OR SU=(弹响髋综合征) OR SU=(髋关节盂唇撕裂) OR SU=(髋关节置换术）OR SU=(hip pain) OR SU=(hip disorder) OR SU=(hip dislocation) OR SU=(hip arthropathy) OR SU=(hip osteoarthritis) OR SU=(hip synovitis) OR SU=(avascular necrosis of femoral head) OR SU=(hip fracture) OR SU=(hip impingement syndrome) OR SU=(iliotibial band syndrome) OR SU=(piriformis syndrome) OR SU=(snapping hip syndrome) OR SU=(hip labrum tear) OR SU=(hip arthrophy)	38156
#9	SU=(随机对照试验临床) OR SU=(随机对照试验) OR SU=(对照临床试验) OR SU=(安慰剂) OR SU=(临床试验) OR SU=(试验)	2787111
#10	SU=(randomized controlled trial) OR SU=(contolled clinical trial)	16589
#11	#9 OR #10 SU=(随机对照试验临床) OR SU=(随机对照试验) OR SU=(对照临床试验) OR SU=(安慰剂) OR SU=(临床试验) OR SU=(试验) OR SU=(randomized controlled trial) OR SU=(contolled clinical trial)	2784299
#12	#5 AND #8 AND #11 (SU=(针) OR SU=(针刺) OR SU=(电针) OR SU=(头皮针) OR SU=(手针) OR SU=(药针) OR SU=(acupuncture) OR SU=(electroacupuncture) OR	21

	SU=(auricular acupuncture) OR SU=(ear acupuncture) OR SU=(scalp acupuncture) OR SU=(hand acupuncture) OR SU=(pharmacoacupuncture)) AND (SU=(髋关节疼痛) OR SU=(髋关节紊乱) OR SU=(髋关节脱位) OR SU=(髋关节病) OR SU=(髋关节骨性关节炎) OR SU=(髋关节滑膜炎) OR SU=(股骨头缺血性坏死) OR SU=(髋部骨折) OR SU=(髋关节撞击综合征) OR SU=(髂胫束综合征) OR SU=(梨状肌综合征) OR SU=(弹响髋综合征) OR SU=(髋关节盂唇撕裂) OR SU=(髋关节置换术) OR SU=(hip pain) OR SU=(hip disorder) OR SU=(hip dislocation) OR SU=(hip arthropathy) OR SU=(hip osteoarthritis) OR SU=(hip synovitis) OR SU=(avascular necrosis of femoral head) OR SU=(hip fracture) OR SU=(hip impingement syndrome) OR SU=(iliotibial band syndrome) OR SU=(piriformis syndrome) OR SU=(snapping hip syndrome) OR SU=(hip labrum tear) OR SU=(hip arthrophy)) AND (SU=(随机对照试验临床) OR SU=(随机对照试验) OR SU=(对照临床试验) OR SU=(安慰剂) OR SU=(临床试验) OR SU=(试验) OR SU=(randomized controlled trial) OR SU=(contolled clinical trial))	

Supplementary Table S5. Search strategy in KISS

NO.	Search strategy	Item
#1	전체 = 침|침술|침구|전침|약침|이침|두침|수지침|	77070
#2	전체 = acupuncture|electroacupuncture|auricular acupuncture|ear acupuncture|scalp acupuncture|hand acupuncture|pharmacoacupuncture	7037
#3	#1 OR #2	79505
#4	전체 = 고관절 통증|고관절증|고관절 점액낭염|고관절 힘줄증|고관절 충돌 증후군|고관절 골절|고관절 탈구|이상근 증후군|대퇴골두 무혈성 괴사|비구순 파열|발음성 고관절|장경인대 증후군	5
#5	전체 = hip pain|hip disorder|hip dislocation|hip arthropathy|hip osteoarthritis|hip synovitis|avascular necrosis of femoral head|hip fracture|hip impingement syndrome|iliotibial band syndrome|piriformis syndrome|snapping hip syndrome|hip labrum tear|hip arthrophy	0
#6	#4 OR #5	0
#7	전체: Randomized Trials|무작위 임상|무작위 시험|Randomized Clinical Trials	1109
#8	#3 AND #5 AND #7	0

supplementary table S6. search strategy in RISS

<table>
<thead>
<tr><th>NO.</th><th>Search strategy</th><th>Item</th></tr>
</thead>
<tbody>
<tr><td>#1</td><td>전체 : 침|침술|침구|전침|약침|이침|두침|수지침|</td><td>68134</td></tr>
<tr><td>#2</td><td>전체 : acupuncture|electroacupuncture|(auricular acupuncture)|(ear acupuncture)|(scalp acupuncture)|(hand acupuncture)|(pharmacoacupuncture)</td><td>37125</td></tr>
<tr><td>#3</td><td>#1 OR #2</td><td>99276</td></tr>
<tr><td>#4</td><td>전체 : (고관절 통증)|(고관절증)|(고관절 점액낭염)|(고관절 힘줄증)|(고관절 충돌증후군)|(고관절 골절)|(고관절 탈구)|(이상근 증후군)|(대퇴골두 무혈성 괴사)|(비구순 파열)|(발음성 고관절)|(장경인대 증후군)</td><td>3161</td></tr>
<tr><td>#5</td><td>전체 : (hip pain)|(hip disorder)|(hip dislocation)|(hip arthropathy)|(hip osteoarthritis)|(hip synovitis)|(avascular necrosis of femoral head)|(hip fracture)|(hip impingement syndrome)|(iliotibial band syndrome)|(piriformis syndrome)|(snapping hip syndrome)|(hip labrum tear)|(hip arthrophy)</td><td>24116</td></tr>
<tr><td>#6</td><td>#4 OR #5

전체 : (고관절 통증)|(고관절증)|(고관절 점액낭염)|(고관절 힘줄증)|(고관절 충돌증후군)|(고관절 골절)|(고관절 탈구)|(이상근 증후군)|(대퇴골두 무혈성 괴사)|(비구순 파열)|(발음성 고관절)|(장경인대 증후군) <OR> 전체 : (hip pain)|(hip disorder)|(hip dislocation)|(hip arthropathy)|(hip osteoarthritis)|(hip synovitis)|(avascular necrosis of femoral head)|(hip fracture)|(hip impingement syndrome)|(iliotibial band syndrome)|(piriformis syndrome)|(snapping hip syndrome)|(hip labrum tear)|(hip arthrophy)</td><td>25830</td></tr>
<tr><td>#7</td><td>전체: (Randomized Trials)|(무작위 임상)|(무작위 시험)|(Randomized Clinical Trials)</td><td>128430</td></tr>
<tr><td>#8</td><td>#3 AND #5 AND #7</td><td>8</td></tr>
</tbody>
</table>

supplementary table S7. search strategy in OASIS

NO.	Search strategy	Item
#1	전체 : 침|침술|침구|전침|약침|이침|두침|수지침	1667
#2	전체 : acupuncture|electroacupuncture|auricular acupuncture|ear acupuncture|scalp acupuncture|hand acupuncture|pharmacoacupuncture	4516
#3	#1 OR #2	4516
#4	전체 : 고관절 통증|고관절증|고관절 점액낭염|고관절 힘줄증|고관절 충돌증후군|고관절 골절|고관절 탈구|이상근 증후군|대퇴골두 무혈성 괴사|비구순 파열|발음성 고관절|장경인대 증후군	1
#5	전체 : hip pain|hip disorder|hip dislocation|hip arthropathy|hip osteoarthritis|hip synovitis|avascular necrosis of femoral head|hip fracture|hip impingement syndrome|iliotibial band syndrome|piriformis syndrome|snapping hip syndrome|hip labrum tear|hip arthrophy	0
#6	#4 OR #5	0
#7	전체: Randomized Trials|무작위 임상|무작위 시험|Randomized Clinical Trials	55
#8	#3 AND #5 AND #7	0
	전체 : (침|침술|침구|전침|약침|이침|두침|수지침 |acupuncture|electroacupuncture|auricular acupuncture|ear acupuncture|scalp acupuncture|hand acupuncture|pharmacoacupuncture) AND (고관절 통증|고관절증|고관절 점액낭염|고관절 힘줄증|고관절 충돌증후군|고관절 골절|고관절 탈구|이상근 증후군|대퇴골두 무혈성 괴사|비구순 파열|발음성 고관절|장경인대 증후군|hip pain|hip disorder|hip dislocation|hip arthropathy|hip osteoarthritis|hip synovitis|avascular necrosis of femoral head|hip fracture|hip impingement syndrome|iliotibial band syndrome|piriformis syndrome|snapping hip syndrome|hip labrum tear|hip arthrophy) AND (Randomized Trials|무작위 임상|무작위 시험|Randomized Clinical Trials)	

supplementary table S8. search strategy in Scienceon

NO.	Search strategy	Item
#1	전체 =침|침술|침구|전침|약침|이침|두침|수지침 |acupuncture|electroacupuncture|auricular acupuncture|ear acupuncture|scalp acupuncture|hand acupuncture|pharmacoacupuncture	1253815
#2	전체 =고관절 통증|고관절증|고관절 점액낭염|고관절 힘줄증|고관절 충돌증후군|고관절 골절|고관절 탈구|이상근 증후군|대퇴골두 무혈성 괴사|비구순 파열|발음성 고관절|장경인대 증후군|hip pain|hip disorder|hip dislocation|hip arthropathy|hip osteoarthritis|hip synovitis|avascular necrosis of femoral head|hip fracture|hip impingement syndrome|iliotibial band syndrome|piriformis syndrome|snapping hip syndrome|hip labrum tear|hip arthrophy	0
#3	전체=Randomized Trials|무작위 임상|무작위 시험|Randomized Clinical Trials	91563
#4	#1 AND #2 AND #3<br><br>전체=침|침술|침구|전침|약침|이침|두침|수지침 |acupuncture|electroacupuncture|auricular acupuncture|ear acupuncture|scalp acupuncture|hand acupuncture|pharmacoacupuncture AND 전체=고관절 통증|고관절증|고관절 점액낭염|고관절 힘줄증|고관절 충돌증후군|고관절 골절|고관절 탈구|이상근 증후군|대퇴골두 무혈성 괴사|비구순 파열|발음성 고관절|장경인대 증후군|hip pain|hip disorder|hip dislocation|hip arthropathy|hip osteoarthritis|hip synovitis|avascular necrosis of femoral head|hip fracture|hip impingement syndrome|iliotibial band syndrome|piriformis syndrome|snapping hip syndrome|hip labrum tear|hip arthrophy AND 전체=Randomized Trials|무작위 임상|무작위 시험|Randomized Clinical Trials	0
